# Supplementary material for: DNA lesions can frequently precede DNA:RNA hybrid accumulation
Source: Nat Commun. 2025 Mar 10;16:2401. doi: 10.1038/s41467-025-57588-x (PMC11893903; doi:10.1038/s41467-025-57588-x)
Supplement: Supplementary file 1 — Supplementary Information [file 41467_2025_57588_MOESM1_ESM.pdf]

SUPPLEMENTARY INFORMATION

DNA lesions can frequently precede DNA:RNA hybrid accumulation

Raphaël M. Mangione, Steven Pierce, Myriam Zheng, Robert M. Martin, Coralie Goncalves, Arun Kumar, Sarah Scaglione, Cristiana de Sousa Morgado, Arianna Penzo, Astrid Lancrey, Robert J.D. Reid, Ophélie Lautier, Pierre-Henri Gaillard, Peter C. Stirling, Sérgio F. de Almeida, Rodney Rothstein & Benoit Palancade

includes:

- Supplementary Fig. 1-8.
- Supplementary Table 1 (Strains used in this study).
- Supplementary Table 2 (Plasmids used in this study).
- Supplementary Table 3 (Oligonucleotides for real-time PCR, synthetic spike-in and *in vitro* cleavage assays).
- Supplementary references.

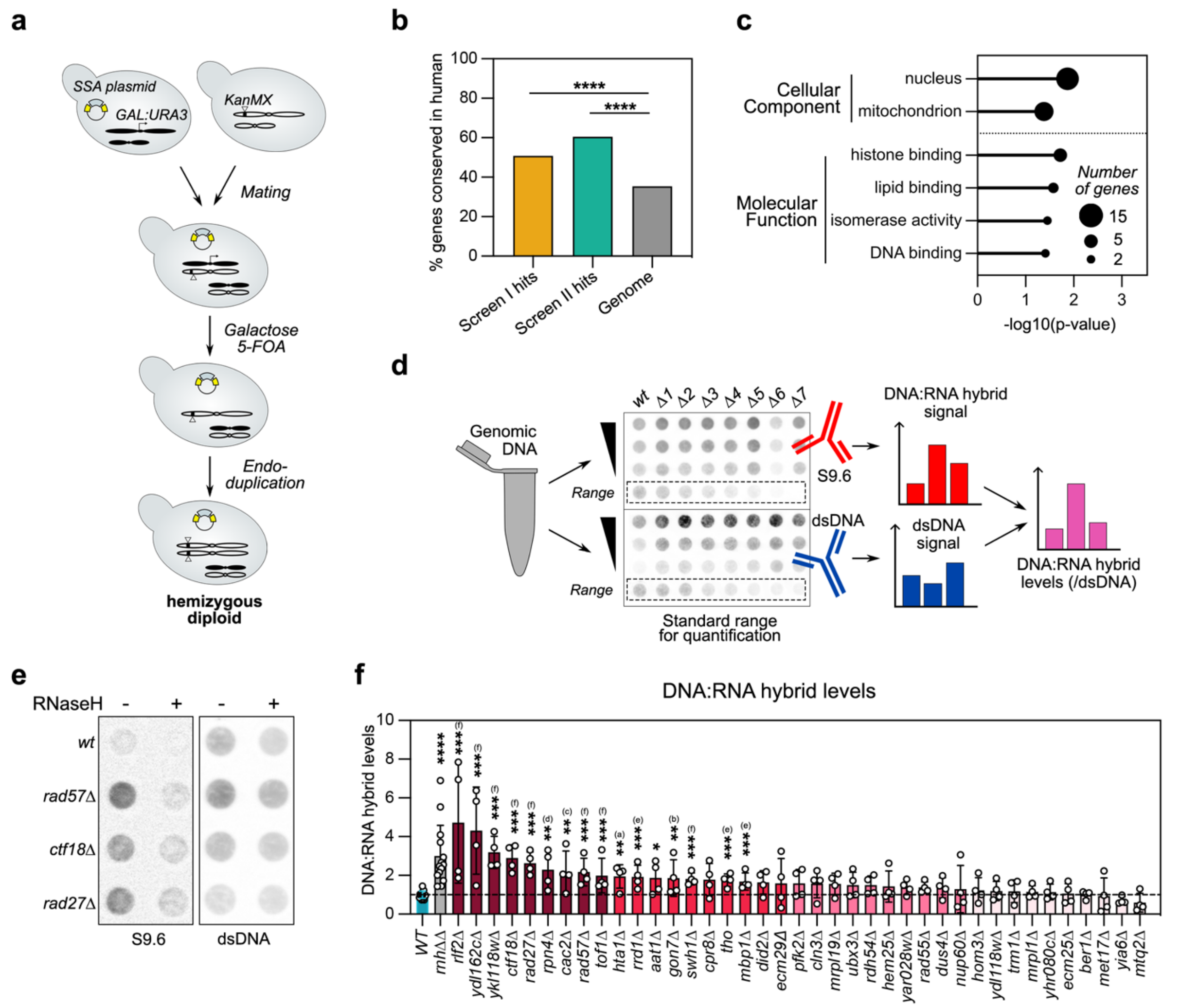

Supplementary Fig. 1. Recombination and DNA:RNA hybrid levels measurements in systematic screens. a, Principle of systematic hybrid loss of heterozygosity[1]. Chromosomes carrying the centromeric *GAL:URA3* allele are destabilized on galactose/5FOA containing medium. **b**, The fraction of genes conserved in human (%) is represented for gene subsets identified in Screen I (n=114; ****, p=2.2x10^{-7}) or Screen II (n=39; ****, p=9.1x10^{-7}). The fraction of yeast genes conserved in human for the whole yeast genome is also indicated. **c**, Gene Ontology analysis (Cellular Component; Molecular Function) for the 39 genes whose deletion triggers significant hyper-recombination in both screens I and II. **d**, Principle of the dot blot assays used in Screen III. Decreasing amounts of genomic DNA are spotted for each mutant on interest, together with negative and positive controls (*wt* and *rnh1*Δ *rnh201*Δ, respectively) and a standard range for quantification. **e**, DNA:RNA hybrid levels were assessed by dot blot on genomic DNA obtained from the indicated strains using S9.6 antibodies (left panel). dsDNA levels were used as loading control (anti-dsDNA, right panel). When indicated, genomic DNA extracts were treated with RNase H *in vitro* prior to dot blotting (RNase H: +). **f**, DNA:RNA hybrid levels were quantified for all 39 hyper-rec mutants commonly found in both screens (I and II) through dot blot analyses performed as in **d** (mean±SD ; *wt*, n=17, *rnh*ΔΔ, n=14, all other mutants, n=4 ; relative to dsDNA and mean control values; *, p=0.0237; **$^{(a)}$, p=0.009; **$^{(b)}$, p=0.006; **$^{(c)}$, p=0.004; **$^{(d)}$, p=0.0013; ***$^{(e)}$, p=0.0007; ***$^{(f)}$, p=0.0003; ****, p<0.0001). The *rnh1*Δ *rnh201*Δ (*rnh*ΔΔ) RNase H double mutant was used as a hybrid-accumulating control.

Statistical tests: **b-c**: Hypergeometric test; **f**, Two-sided Mann-Whitney-Wilcoxon rank sum test. Source data are provided as a Source Data file.

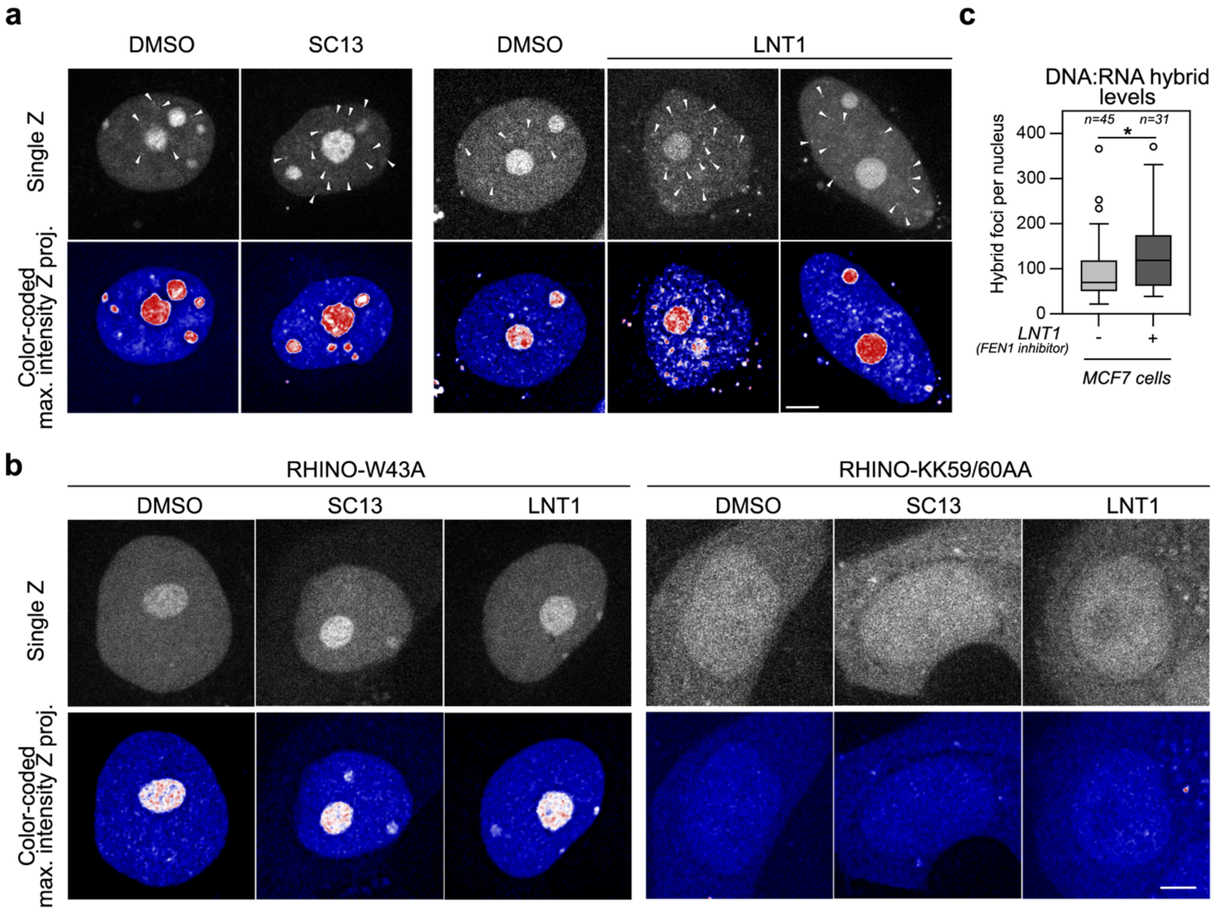

Supplementary Fig. 2. FEN1 inhibition triggers DNA:RNA hybrid accumulation in human cells. **a-b,** DNA:RNA hybrid detection in live MCF7 cells expressing either WT (**a**), W43A (**b**) or KK59/60AA (**b**) RHINO sensors. When indicated, cells were treated with FEN1 inhibitors (SC13 or LNT1; 50 µM) or an equivalent volume of DMSO as control. The images show representative single confocal z-sections (top panels) and filtered maximum intensity projections of the same cell nuclei in a color-coded intensity display to highlight RHINO foci (bottom panels). Note the absence of detectable hybrid foci (as pointed by arrowheads in panel **a**) upon expression of RHINO mutant versions defective for hybrid binding (W43A, KK59/60AA; panel **b**). Scale bar, 5 µm. **c,** Quantification of DNA:RNA hybrid foci in MCF7 breast cancer cells expressing the RHINO sensor and further treated with the LNT1 FEN1 inhibitor (n=3; total number of cells: control, n=45, LNT1, n= 31; *, p=0.0145). Boxes extend from the 25th to 75th percentiles, with the median displayed as a line. The whiskers mark 1.5 time the inter-quartile range of the first or third quartile (Tukey's definition), displaying outliers as individual points.
Statistical test: Two-sided Mann-Whitney-Wilcoxon rank sum test. Source data are provided as a Source Data file.

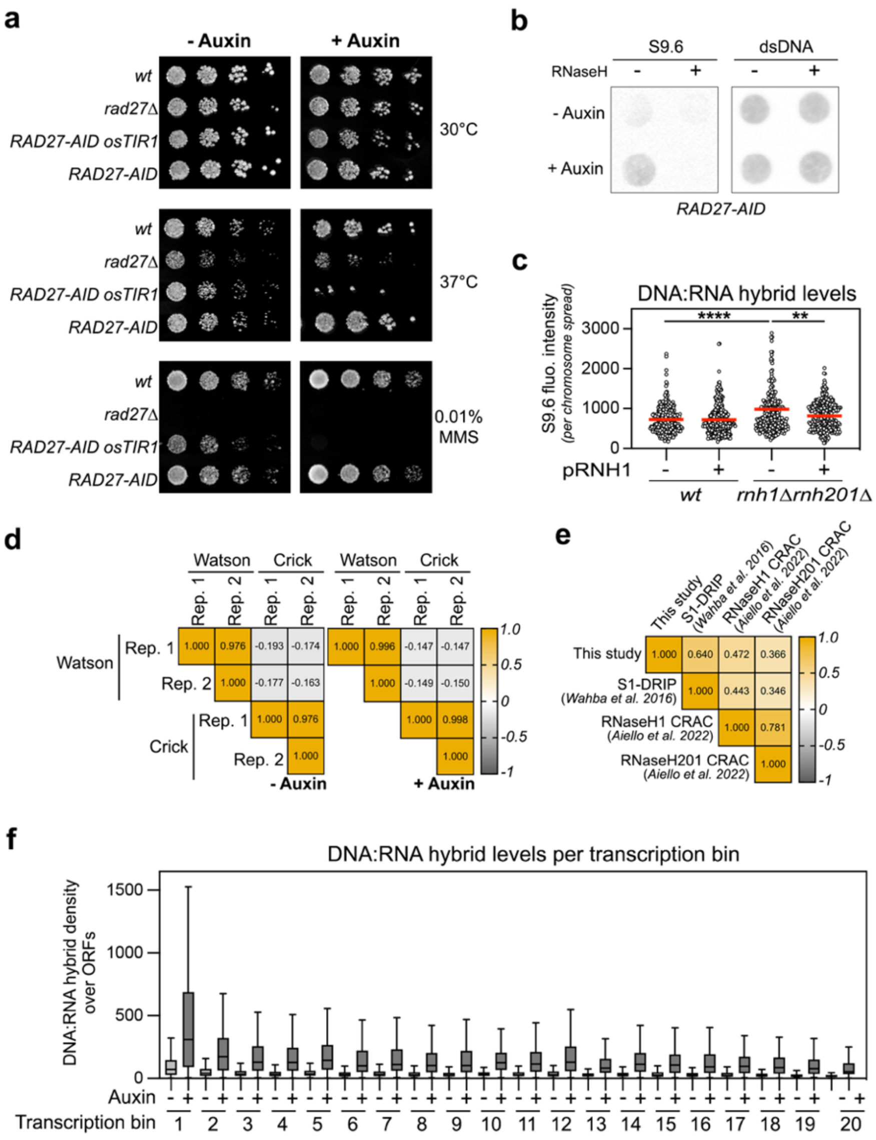

Supplementary Fig. 3. Genome-wide analysis of DNA:RNA hybrid distribution upon Rad27 depletion. a, Serial dilutions of the indicated strains were grown at the indicated temperatures (30°C, 37°C) on rich medium (YPD). When indicated, media contained auxin or methylmethane sulfonate (MMS). **b**, DNA:RNA hybrid levels were assessed by dot blot on genomic DNA obtained from *RAD27-AID* cells, either control or treated with auxin, using S9.6 (left panel) and anti-dsDNA antibodies (right panel). When indicated, genomic DNA extracts were treated with RNase H *in vitro* prior to dot blotting (RNase H: +). **c**, Positive control for the chromosome spreads experiment (**Fig. 2e**). DNA:RNA hybrid levels (n=3; total number of cells: *wt*, n=306, *wt pRNH1*, n=280, *rnh1Δ rnh201Δ*, n=316, *rnh1Δ rnh201Δ pRNH1,* n=300; **, p=0.0024; ****, p<0.0001) were assessed by immunofluorescence using S9.6 antibodies on chromosome spreads obtained from *rnh1Δ rnh201Δ* cells. When indicated, cells carried a plasmid allowing ectopic ScRNH1 expression (*pRNH1,* +). *wt* values are the same as in **Fig. 2e**. **d,** Correlation analysis for DRIP-seq biological replicates (Rep. 1 and Rep. 2), comparing signal from Watson or Crick strands. For each comparison, the Pearson correlation coefficient is indicated. **e**, Correlation analysis for distinct DNA:RNA hybrid datasets obtained from control cells: strand-specific DRIP-seq (*RAD27-AID*, minus auxin ; this study); S1-DRIP-seq (*wt* cells, from Wahba et al.[2]) ; RNase H CRAC (using tagged Rnh1 or Tagged Rnh201 in *wt* cells, from Aiello et al.[3]). **f**, RNAP II-transcribed regions were split into 20 categories according to their level of transcription[4], from the most (bin #1) to the least (bin #20) expressed. For each transcription bin, DNA:RNA hybrid levels densities over transcribed regions (TSS-TES) were displayed for control (auxin: -) or Rad27-depleted (auxin: +) conditions. Box-plots are defined as above (**Supplementary Fig. 2c**).

Statistical test: Two-sided Mann-Whitney-Wilcoxon rank sum test. Source data are provided as a Source Data file.

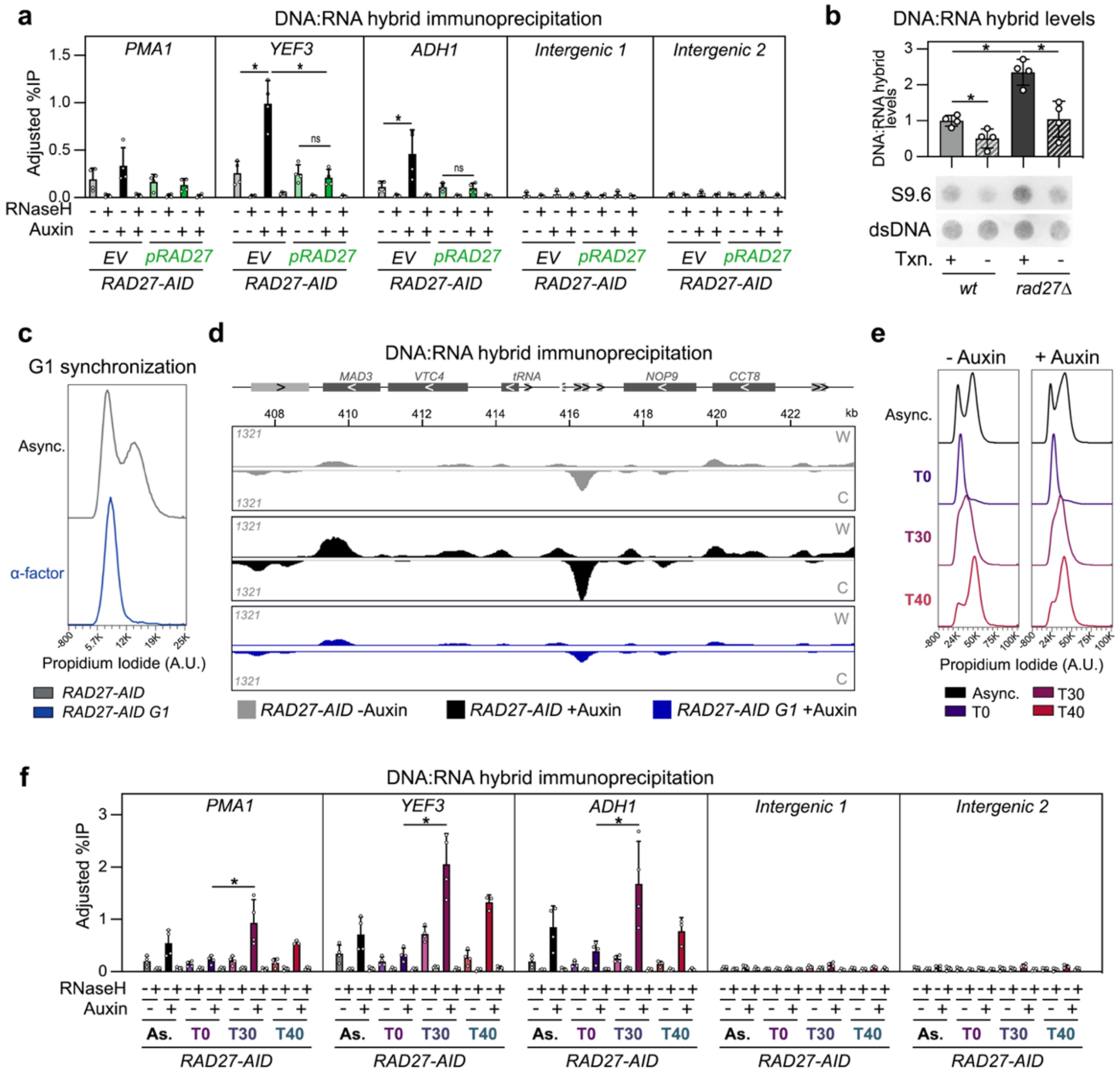

Supplementary Fig. 4. Transcription- and replication-dependent DNA:RNA hybrid accumulation upon Rad27 depletion. a, DNA:RNA hybrid detection (DRIP-qPCR; adjusted % of IP; mean±SD; n=4; *, p=0.0286) at the indicated loci in *RAD27-AID* cells either control (auxin: -) or treated with auxin (auxin: +). Cells carried either an empty vector (*EV*) or a complementing construct expressing the Rad27 *wt* protein (*pRAD27*). When indicated, DNA extracts were treated with RNase H *in vitro* prior to immunoprecipitation (RNase H: +). EV values are the same as in **Fig. 3f** (*YEF3*) and **Supplementary Fig. 5b** (*PMA1, ADH1,* intergenic regions). **b**, DNA:RNA hybrid levels were assessed by dot blot on genomic DNA obtained from *wt* or *rad27*Δ cells, either control (Txn.:+) or treated with the 1,10-phenanthroline transcriptional inhibitor (mean±SD; n=4; relative to dsDNA and mean control values; *, p=0.0286). **c**, Flow cytometry analysis of *RAD27-AID* cells grown in the presence or absence of alpha-factor and labeled with propidium iodide. **d,** Integrative Genomics Viewer (IGV) representative screenshots of DRIP-seq coverage in *RAD27-AID* cells either control, treated with auxin, or arrested in G1 prior to auxin treatment. Signals from Watson (W) or Crick (C) strands are displayed for a representative replicate of DRIP immunoprecipitates. **e**, Flow cytometry analysis of *RAD27-AID* cells, either asynchronous (Async.), or synchronized in G1 (T0) and released into the cell cycle for 30 or 40 min (T30, T40). Cells were labeled with propidium iodide. **f**, DNA:RNA hybrid detection (DRIP-qPCR; adjusted % of IP; mean±SD; n=4 for Async., T0, T30, and n=3 for T40; *, p=0.0286) at the indicated loci in *RAD27-AID* cells either control (auxin: -) or treated with auxin (auxin: +). Cell cultures were either asynchronous (Async.), or synchronized in G1 and released into the cell cycle for the indicated durations (T30, 30 min; T40, 40 min). When indicated, DNA extracts were treated with RNase H *in vitro* prior to immunoprecipitation (RNase H: +).

Statistical test: Two-sided Mann-Whitney-Wilcoxon rank sum test. Source data are provided as a Source Data file.

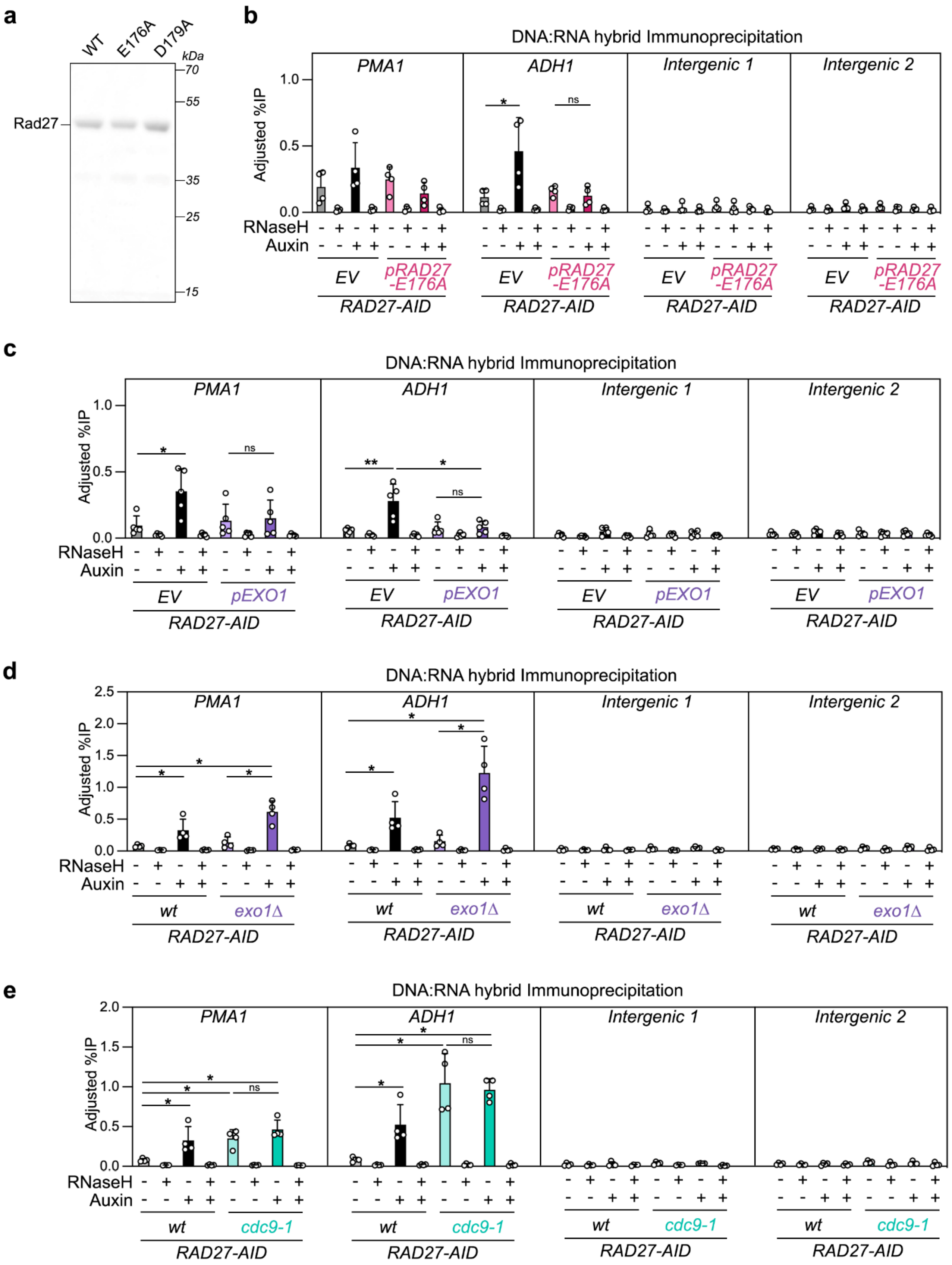

Supplementary Fig. 5. DNA discontinuities associated with OF processing defects precede DNA:RNA hybrid accumulation. See legend on next page.

Supplementary Fig. 5. DNA discontinuities associated with OF processing defects precede DNA:RNA hybrid accumulation.

a, Purified preparations of Rad27 recombinant proteins, either *wt*, D179A or E176A, were analyzed by SDS-PAGE followed by Coomassie blue staining. Molecular weights are indicated (kDa; Rad27-His6 expected size: 44 kDa). **b**, DNA:RNA hybrid detection (DRIP-qPCR; adjusted % of IP; mean±SD; n=4; *, p=0.0286) at the indicated loci in *RAD27-AID* cells either control (auxin: -) or treated with auxin (auxin: +). Cells carried either an empty vector (*EV*) or a construct expressing the Rad27-E176A mutant protein (*pRAD27-E176A).* **c-e**, DNA:RNA hybrid detection (DRIP-qPCR; adjusted % of IP; mean±SD; **c**, n=5, *, p=0.0159; **, p=0.0079; **d-e**, n=4, *, p=0.0286) at the indicated loci in *RAD27-AID wt, exo1*Δ (**d**) or *cdc9-1* (**e**;) derivatives, either control (auxin: -) or treated with auxin (auxin: +). In panel **c**, cells carried either an empty vector (*EV*) or a construct over-expressing Exo1 (*pEXO1*). The same *wt* control is used in panels **d** and **e**.

For all DRIP-qPCR experiments, when indicated, DNA extracts were treated with RNase H *in vitro* prior to immunoprecipitation (RNase H: +). Statistical test: Two-sided Mann-Whitney-Wilcoxon rank sum test. Source data are provided as a Source Data file.

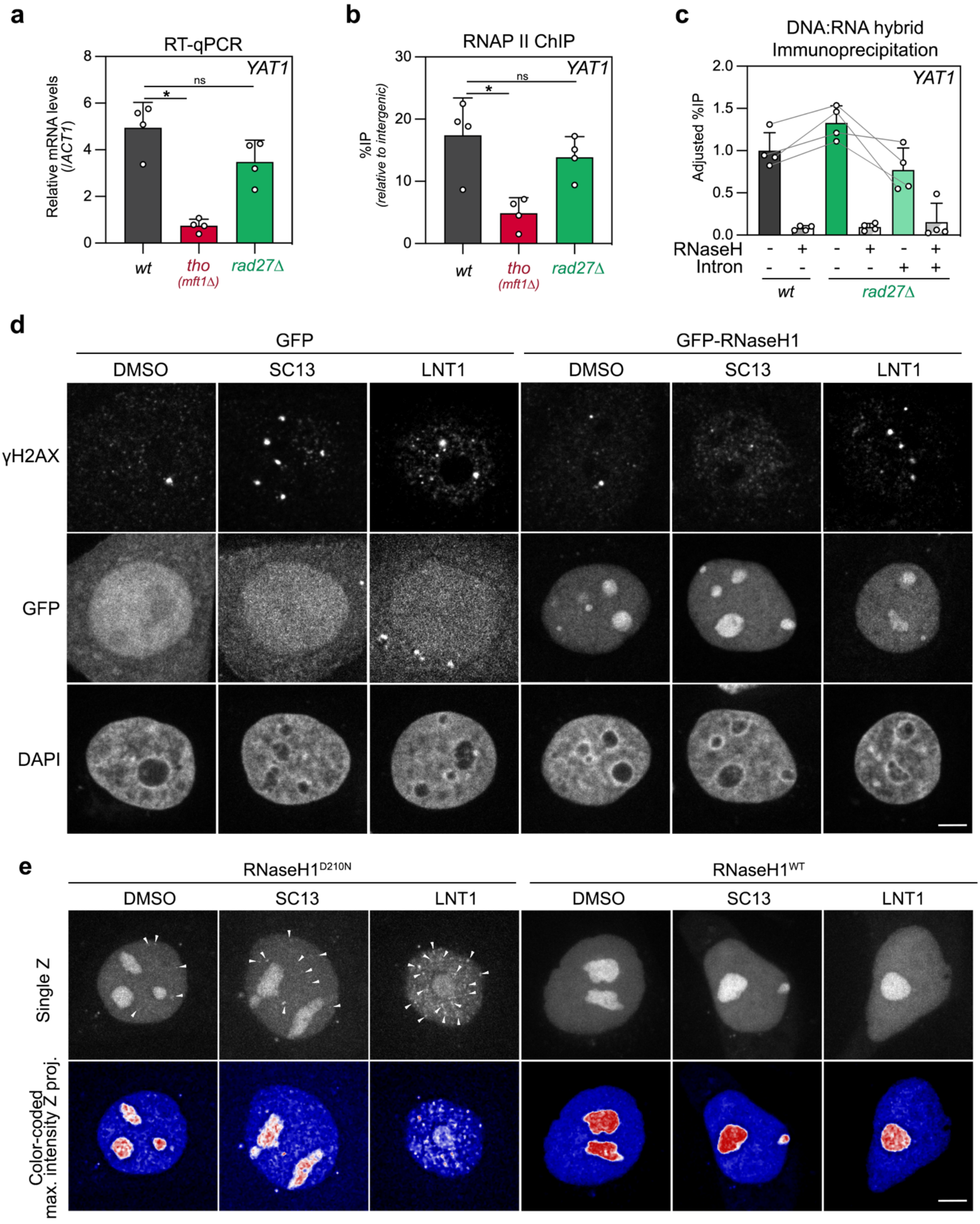

Supplementary Fig. 6. Post-lesion DNA:RNA hybrids do not compromise genome expression or stability. See legend on next page.

Supplementary Fig. 6. Post-lesion DNA:RNA hybrids do not compromise genome expression or stability.

a, Real time PCR quantification of *YAT1* mRNA levels from the indicated strains carrying the *GAL-YAT1* transgene, grown in glycerol-lactate medium and further treated with galactose for 5 h (values normalized to *ACT1* mRNA levels ; mean±SD, n=4; *, p=0.0286). **b,** RNAP II occupancy was analyzed at the *GAL-YAT1* transgene by ChIP and real time PCR in the indicated strains treated with galactose for 5 h (% of immunoprecipitation, relative to intergenic; mean±SD, n=4; *, p=0.0286). **c**, DNA:RNA hybrid detection (DRIP-qPCR; adjusted % of IP; mean±SD; n=4) at *YAT1* transgenes in cells of the indicated genotypes carrying *GAL1-YAT1* or *GAL-intron-YAT1* (Intron: +) constructs, grown in glycerol-lactate medium and further treated with galactose for 30 min. When indicated, DNA extracts were treated with RNase H *in vitro* prior to immunoprecipitation (RNase H: +). **d**, Immunofluorescence analysis of DNA damage (γH2AX) in MCF7 cells transfected with GFP-RNase H1 or control (GFP) constructs, and further incubated for 24 h with either FEN1 inhibitors (SC13 or LNT1, 50 μM) or DMSO as control. Direct GFP fluorescence and DNA staining (DAPI) are also shown. Scale bar, 5 μm. **e**, DNA:RNA hybrid detection in live MCF7 cells expressing either wild type (wt) or catalytic dead (D210N) RNaseH1. When indicated, cells were treated with FEN1 inhibitors (SC13 or LNT1; 50 μM) or an equivalent volume of DMSO as control. The images show representative single confocal z-sections (top panels) and filtered maximum intensity projections of the same cell nuclei in a color-coded intensity display to highlight RHINO foci (bottom panels). Note the absence of detectable hybrid foci (as pointed by arrowheads) upon expression of wt RNase H1. Scale bar, 5 μm.

Statistical test: Two-sided Mann-Whitney-Wilcoxon rank sum test. Source data are provided as a Source Data file.

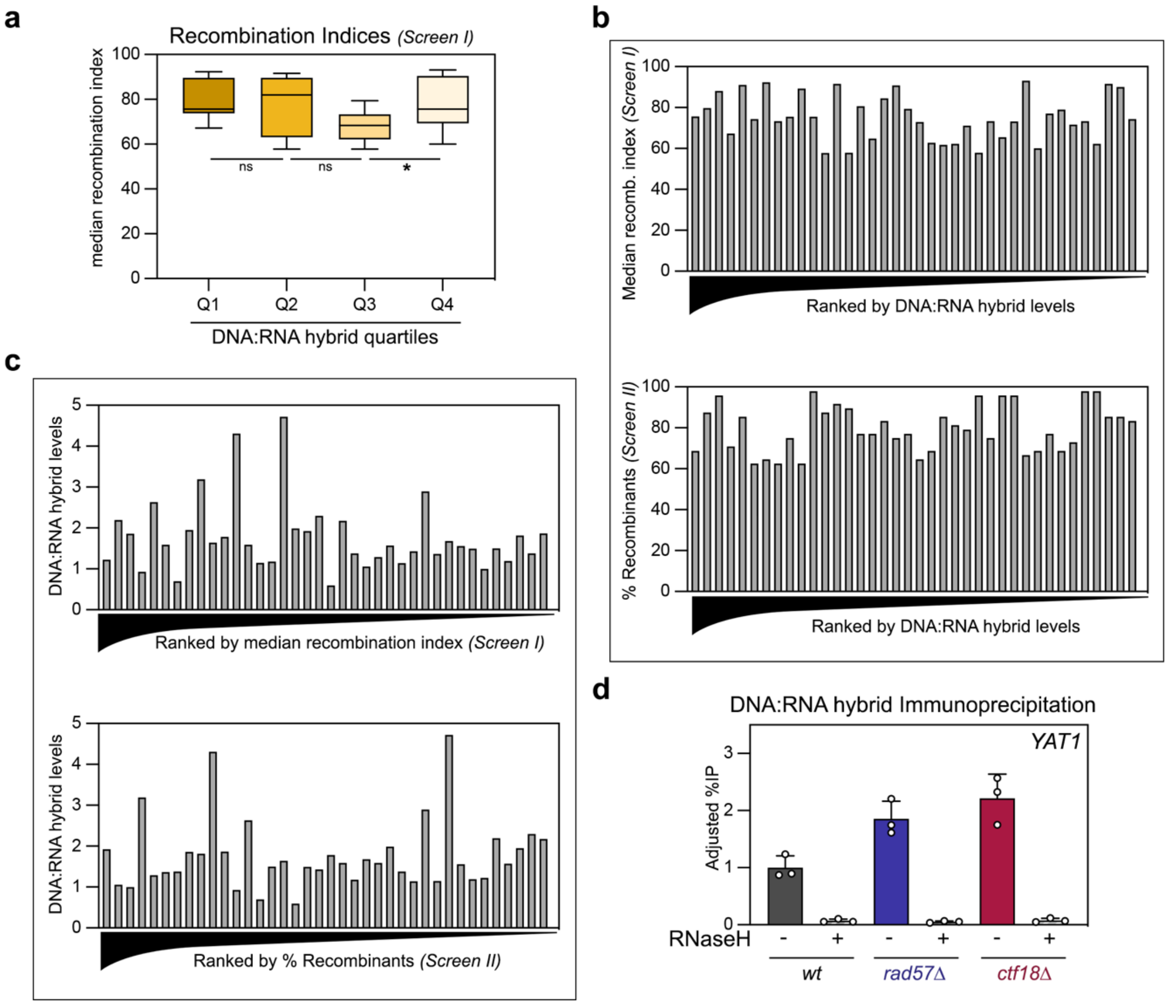

Supplementary Fig. 7. Genetic instability does not generally correlate with DNA:RNA hybrid accumulation in hyper-recombinant mutants. a, Hyper-rec mutants common to Screen I and II (n=39) were split into quartiles according to DNA:RNA hybrid levels and recombination indices from screen I are represented for each quartile (*, p=0.0440). Box-plots are defined as above (**Supplementary Fig. 2c**). **b**, Median recombination indices (from screen I, top panel) and recombination levels (% of *LEU*+ recombinants from screen II, bottom panel) were represented for all 39 hyper-rec mutants commonly found in both screens and ranked by DNA:RNA hybrid levels (as in **Fig. 4h**). **c**, Mean DNA:RNA hybrid levels (from **Supplementary Fig. 1f**) are represented for all 39 hyper-rec mutants commonly found in both screens (I and II), either ranked by median recombination indices (from screen I, top panel) or recombination levels (% of *LEU*+ recombinants from screen II, bottom panel). **d**, DNA:RNA hybrid detection (DRIP-qPCR; adjusted % of IP; mean±SD; n=3) at *YAT1* transgenes in cells of the indicated genotypes, carrying the *GAL1-YAT1* construct and grown as for **Supplementary Fig. 6c**. When indicated, DNA extracts were treated with RNase H *in vitro* prior to immunoprecipitation (RNase H: +). Statistical test: Two-sided Mann-Whitney-Wilcoxon rank sum test. Source data are provided as a Source Data file.

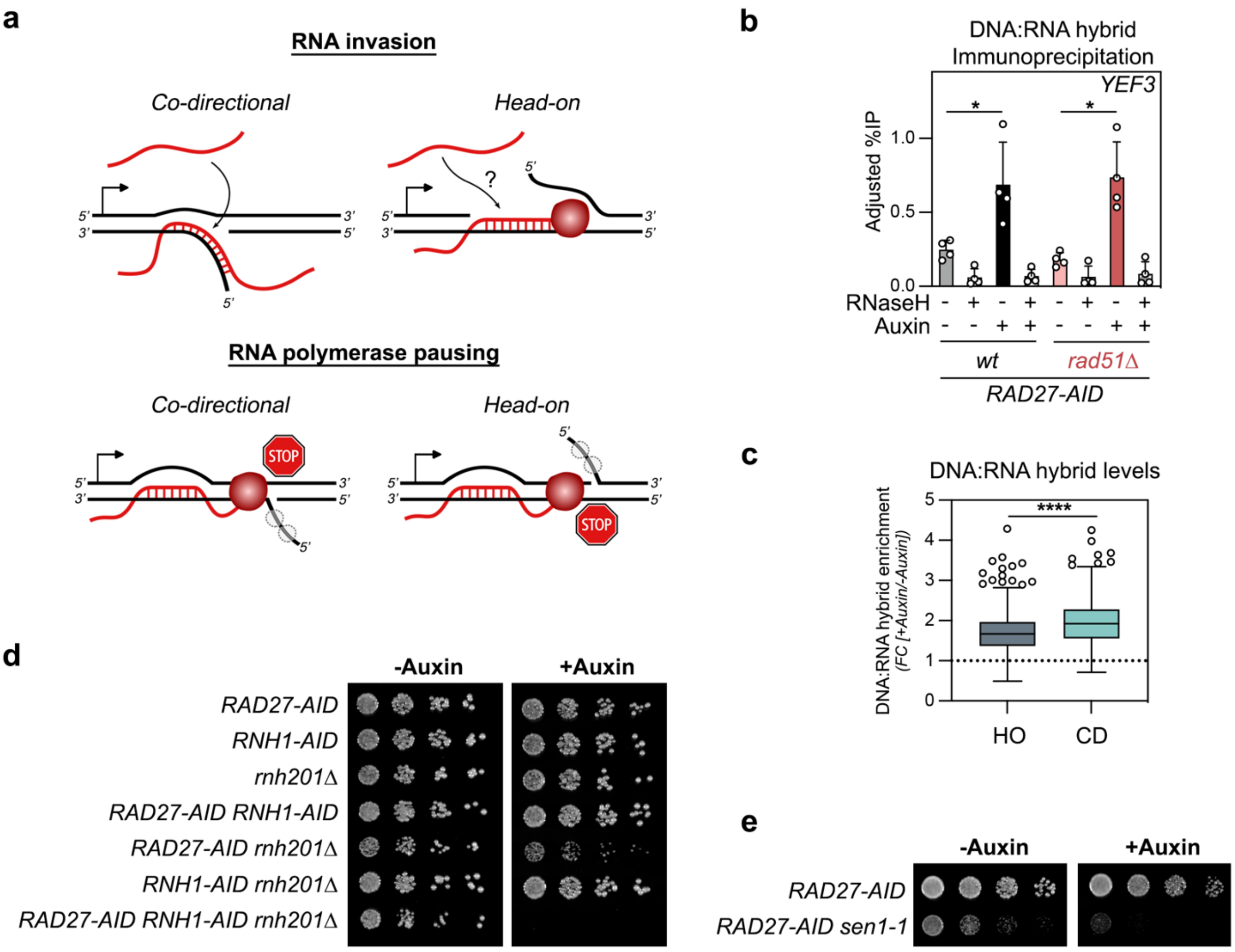

Supplementary Fig. 8. Possible mechanisms of post-lesion hybrid formation and genotoxicity. a, Mechanistic models for the formation of post-lesion DNA:RNA hybrids upon OF processing defects. In the RNA invasion model (top panels), discontinuities such as flaps, SSBs or gaps would allow the invasion of homologous RNA molecules, thus forming DNA:RNA hybrids. Depending on the orientation of the replication fork with respect to the transcription unit, hybrid-forming RNAs could originate from previous or concomitant transcription events. In the pausing model (bottom panels), RNA polymerases could stall upon encountering flaps of SSBs on the template or non-template strands, thus favoring upstream hybrid formation. **b,** DNA:RNA hybrid detection (DRIP-qPCR; adjusted % of IP; mean±SD; n=4; *, p=0.0286) by DRIP-qPCR at the *YEF3* locus in *RAD27-AID wt* or *rad51Δ* derivatives, either control (auxin: -) or treated with auxin (auxin: +). When indicated, DNA extracts were treated with RNase H *in vitro* prior to immunoprecipitation (RNase H: +). **c,** Quantification of DNA:RNA hybrid enrichment following Rad27 depletion (Fold Change + Auxin / - Auxin) on genes transcribed either in co-directional (CD) or head-on (HO) orientation relative to replication fork progression (****, p<0.0001), as inferred from OK-seq analyses (*wt* cells[5]). Box-plots are defined as above (**Supplementary Fig. 2c**). **d-e**, Serial dilutions of the indicated strains were grown at 30°C on rich medium (YPD), either control, or supplemented with auxin, as indicated.
Statistical test: Two-sided Mann-Whitney-Wilcoxon rank sum test. Source data are provided as a Source Data file.

Supplementary Table 1 (Strains used in this study)

CODE	NAME	GENOTYPE	SOURCE
Saccharomyces cerevisiae			
yBP539	*wt (BY4742)*	MATalpha *ura3 his3 leu2 lys2*	
yBP936	*wt (BY4741)*	MATa *ura3 his3 leu2 met15*	
YKO mat alpha collection	*x*Δ	(BY4742) *x::KanMX*	Saccharomyces Genome Deletion Project
DY6281	*CEN1::GAL-K.l.URA3*	MATa *ade2 can1 ura3 his3 leu2 trp1 lys2 met15 CEN1::GAL-K.l.URA3*	Reid et al.[6]
W3616-3C	*CEN2::GAL-K.l.URA3*	MATa *ade2 can1 ura3 his3 leu2 trp1 lys2 met15 CEN2::GAL-K.l.URA3*	Reid et al.[6]
DY6280	*CEN3::GAL-K.l.URA3*	MATa *ade2 can1 ura3 his3 leu2 trp1 lys2 met15 CEN3::GAL-K.l.URA3*	Reid et al.[6]
DY6282	*CEN4::GAL-K.l.URA3*	MATa *ade2 can1 ura3 his3 leu2 trp1 lys2 met15 CEN4::GAL-K.l.URA3*	Reid et al.[6]
DY6283	*CEN5::GAL-K.l.URA3*	MATa *ade2 can1 ura3 his3 leu2 trp1 lys2 met15 CEN5::GAL-K.l.URA3*	Reid et al.[6]
DY6284	*CEN6::GAL-K.l.URA3*	MATa *ade2 can1 ura3 his3 leu2 trp1 lys2 met15 CEN6::GAL-K.l.URA3*	Reid et al.[6]
DY6285	*CEN7::GAL-K.l.URA3*	MATa *ade2 can1 ura3 his3 leu2 trp1 lys2 met15 CEN7::GAL-K.l.URA3*	Reid et al.[6]
DY6286	*CEN8::GAL-K.l.URA3*	MATa *ade2 can1 ura3 his3 leu2 trp1 lys2 met15 CEN8::GAL-K.l.URA3*	Reid et al.[6]
DY6287	*CEN9::GAL-K.l.URA3*	MATa *ade2 can1 ura3 his3 leu2 trp1 lys2 met15 CEN9::GAL-K.l.URA3*	Reid et al.[6]
DY6288	*CEN10::GAL-K.l.URA3*	MATa *ade2 can1 ura3 his3 leu2 trp1 lys2 met15 CEN10::GAL-K.l.URA3*	Reid et al.[6]
DY6289	*CEN11::GAL-K.l.URA3*	MATa *ade2 can1 ura3 his3 leu2 trp1 lys2 met15 CEN11::GAL-K.l.URA3*	Reid et al.[6]
DY6290	*CEN12::GAL-K.l.URA3*	MATa *ade2 can1 ura3 his3 leu2 trp1 lys2 met15 CEN12::GAL-K.l.URA3*	Reid et al.[6]
DY6291	*CEN13::GAL-K.l.URA3*	MATa *ade2 can1 ura3 his3 leu2 trp1 lys2 met15 CEN13::GAL-K.l.URA3*	Reid et al.[6]
W3617-1A	*CEN14::GAL-K.l.URA3*	MATa *ade2 can1 ura3 his3 leu2 trp1 lys2 met15 CEN14::GAL-K.l.URA3*	Reid et al.[6]
DY6293	*CEN15::GAL-K.l.URA3*	MATa *ade2 can1 ura3 his3 leu2 trp1 lys2 met15 CEN15::GAL-K.l.URA3*	Reid et al.[6]
DY6294	*CEN16::GAL-K.l.URA3*	MATa *ade2 can1 ura3 his3 leu2 trp1 lys2 met15 CEN16::GAL-K.l.URA3*	Reid et al.[6]
W8164-2C	*MATa superstrain*	MATa *ura3 his3 leu2 trp1 CEN1-16::GAL-K.l.URA3*	Reid et al.[7]
yBP2200	*rad27*Δ	(BY4741) *rad27::KanMX*	Saccharomyces Genome Deletion Project
yBP2239	*RAD27-FLAG-AID osTIR1*	(BY4741) *RAD27-FLAG-AID::KanMX::ADH1*$_{prom}$*-osTIR1*	This study (a).
yBP2317	*RAD27-FLAG-AID*	(BY4741) *RAD27-FLAG-AID::NatMX*	This study (b).
Y05129	*cdc9-1*	(BY4741) *cdc9-1::KanMX*	Li et al.[8]
yBP2363	*RAD27-FLAG-AID exo1*Δ	(BY4741) *RAD27-FLAG-AID::KanMX::ADH1*$_{prom}$*-osTIR1 exo1::NatMX*	This study (c).
yBP2378	*RAD27-FLAG-AID cdc9-1*	(BY4741) *RAD27-FLAG-AID::KanMX::ADH1*$_{prom}$*-osTIR1 cdc9-1::KanMX*	This study (a).
yBP536	*RAD52-YFP*	MATa *ura3 his3 leu2 trp1 bar1::LEU2 RAD52-YFP*	This study (d).
yBP2460	*RAD27-FLAG-AID RAD52-YFP*	MATa *ura3 his3 leu2 trp1 RAD27-FLAG-AID::KanMX:: ADH1*$_{prom}$*-osTIR1 RAD52-YFP*	This study (e).
yBP2464	*ctf18*Δ	(BY4742) *ctf18::KanMX*	Saccharomyces Genome Deletion Project
yBP2465	*rad57*Δ	(BY4742) *rad57::KanMX*	Saccharomyces Genome Deletion Project
yBP2480	*RAD27-FLAG-AID rad51*Δ	(BY4741) *RAD27-FLAG-AID::KanMX::ADH1*$_{prom}$*-osTIR1 rad51::KanMX*	This study (f).
yBP2305	*RAD27-AID rnh201*Δ	(BY4741) *RAD27-FLAG-AID::KanMX::ADH1*$_{prom}$*-osTIR1 rnh201::NatMX*	This study (g)
yBP2487	*RNH1-AID*	MATa *ura3 his3 leu2 trp1 bar1::LEU2 RNH1-FLAG-AID::HphMX:: ADH1*$_{prom}$*-osTIR1 RAD52-YFP*	This study (h)
yBP2494	*RAD27-AID*	MATalpha *his3 leu2 trp1 ura3 bar1::LEU2 RAD27-FLAG-AID::KanMX:: ADH1*$_{prom}$*-osTIR1 RAD52-YFP*	This study (i).

yBP2495	*RNH1-AID*	MATalpha *his3 leu2 trp1 ura3 bar1::LEU2 RNH1-FLAG-AID::HphMX:: ADH1$_{prom}$-osTIR1 RAD52-YFP*	This study (i).
yBP2507	*rnh201Δ*	MATalpha *ade2 his3 leu2 trp1 ura3 bar1::LEU2 rnh201::NatMX RAD52-YFP*	This study (i).
yBP2492	*RAD27-AID RNH1-AID*	MATa *his3 leu2 ura3 bar1::LEU2 RAD27-FLAG-AID::KanMX:: ADH1$_{prom}$-osTIR1 RNH1-FLAG-AID::HphMX:: ADH1$_{prom}$-osTIR1 RAD52-YFP*	This study (i).
yBP2491	*RAD27-AID rnh201Δ*	MATa *his3 leu2 trp1 ura3 bar1::LEU2 RAD27-FLAG-AID::KanMX:: ADH1$_{prom}$-osTIR1 rnh201::NatMX RAD52-YFP*	This study (i).
yBP2490	*RNH1-AID rnh201Δ*	MATa *his3 leu2 trp1 ura3 bar1::LEU2 RNH1-FLAG-AID::HphMX:: ADH1$_{prom}$-osTIR1 rnh201::NatMX RAD52-YFP*	This study (i).
yBP2493	*RAD27-AID RNH1-AID rnh201Δ*	MATalpha *ade2 his3 leu2 trp1 ura3 bar1::LEU2 RAD27-FLAG-AID::KanMX:: ADH1$_{prom}$-osTIR1 RNH1-FLAG-AID::HphMX:: ADH1$_{prom}$-osTIR1 rnh201::NatMX RAD52-YFP*	This study (i).
yBP2295	*RAD27-AID sen1-1*	*(BY4741) RAD27-FLAG-AID::KanMX::ADH1$_{prom}$-osTIR1 sen1-1::KanMX*	This study (j).
Candida glabrata			
yBPCG2	*hpr1Δ*	*his3 trp1 ura3 hpr1::TRP1*	Bonnet et al.[9]

(a) A recombinogenic cassette encompassing a 3xFlag, the Auxin-Induced Degron, a *KanMX* marker and the *ADH1$_{prom}$-osTIR1* transgene was integrated at *RAD27* 3'UTR (chromosomal locus) in BY4741 or Y05129 strains, thus generating yBP2239 and yBP2378 strains.

(b) The *pADH1-osTIR1* transgene was removed from yBP2239 by homologous recombination with a *NatMX* cassette amplified from pFA6a-NatMX6.

(c) *EXO1* complete CDS was deleted in yBP2239 by homologous recombination with a *NatMX* cassette amplified from pFA6a-NatMX6.

(d) Derived from UM74-3B (*RAD52-YFP,* MATalpha)[10] by successive crosses.

(e) Obtained by crossing yBP536 and yBP2239.

(f) Obtained by crossing yBP2239 and *rad51Δ* (YKO mat alpha collection).

(g) *RNH201* complete CDS was deleted in yBP2239 by homologous recombination with a *NatMX* cassette amplified from pFA6a-NatMX6.

(h) A recombinogenic cassette encompassing a 3xFlag, the Auxin-Induced Degron, a *HphMX* marker (from pFA6a-HphMX6) and the *ADH1$_{prom}$-osTIR1* transgene was integrated at *RNH1* 3'UTR (chromosomal locus) in the yBP536 strain.

(i) Obtained by crossing *RNH1-AID* (yBP2487) and *RAD27-AID rnh201Δ* (yBP2305).

(j) Obtained by crossing yBP2239 and Y12015 (*sen1-1*[8]).

Supplementary Table 2 (Plasmids used in this study)

CODE	NAME	GENOTYPE	USAGE (Fig.)	SOURCE
pBP679	pFA6a-NatMX6	AmpR; *TEF_{prom}-NatMX6-TEF_{term}*	Strain construction	Hentges et al.[11]
pBP672	pFA6a-HphMX6	AmpR; *TEF_{prom}-HphX6-TEF_{term}*	Strain construction	Hentges et al.[11]
pBP2122	pFlag-AID-KanMX	AmpR; *3xFLAG-AID-$ADH1_{term}$::KanMX*	Strain construction	A gift from D. Libri
pBP2123	pKanMX-$ADH1_{prom}$-OsTIR1	AmpR; *KanMX::$ADH1_{prom}$-osTIR1*	Strain construction	A gift from D. Libri
pWJ1699	p-yfp-LYS2	AmpR; *$ADH1_{prom}$-yfpΔ3'::$LYS2_{prom}$-LYS2::5'fs-yfp-$ADH1_{term}$*	Fig. 1a-f, Supplementary Fig. 1b-c, 7a-c (Hyper-rec screen I)	This study (a).
pBP2194	p-leu2-YAT1	AmpR; CEN ; *$LEU2_{prom}$-leu2Δ3'-$GAL1_{prom}$-YAT1::HphMX-leu2Δ5'*	Fig. 1a, d, e, g, 4i, Supplementary Fig. 7b-c (Hyper-rec screen II)	This study (b).
	pMK232-TRE3GS-RHINO-TetON-Puro	AmpR; *$TRE3GS_{prom}$-RHINO ; PGK_{prom}-TetOn(R)3G ; $SV40_{prom}$-PuroR*	Fig. 1j, Supplementary Fig. 2a,c	This study (c)
	pMK232-TRE3GS-RHINO(W43A)-TetON-Puro	AmpR; *$TRE3GS_{prom}$-RHINO(W43A) ; PGK_{prom}-TetOn(R)3G ; $SV40_{prom}$-PuroR*	Supplementary Fig. 2b	Martin et al.[12]
	pMK232-TRE3GS-RHINO(KK59/60AA)-TetON-Puro	AmpR; *$TRE3GS_{prom}$-RHINO(KK59/60AA) ; PGK_{prom}-TetOn(R)3G ; $SV40_{prom}$-PuroR*	Supplementary Fig. 2b	Martin et al.[12]
	pUBC-GFP-C1	*KanR; Ubiquitin C_{prom}-GFP*	Fig. 4g; Supplementary Fig. 6d	Vitor et al.[13]
	pUBC-GFP-RNase H1 (WT)	*KanR; Ubiquitin C_{prom}-GFP-RNAse H1*	Fig. 4g; Supplementary Fig. 6d	This study (d)
	pUBC-mScarlet-i-C1	*KanR; Ubiquitin C_{prom}-mScarlet-i*	Supplementary Fig. 6e	Martin et al.[12]
	pUBC-mScarlet-i-RNase H1 (WT)	*KanR; Ubiquitin C_{prom}-mScarlet-i-RNase H1*	Supplementary Fig. 6e	Martin et al.[12] (d)
	pUBC-mScarlet-i-RNase H1 (D210N)	*KanR; Ubiquitin C_{prom}-mScarlet-i-RNase H1(D210N)*	Supplementary Fig. 6e	Martin et al.[12] (d)
pBP2274	pRS316-RNH1	AmpR; CEN; *URA3* ; *$RNH1_{prom}$-RNH1*	Fig. 2e, Supplementary Fig. 3c	Amon and Koshland[14]
pBP2125	pRS316-RAD27	AmpR; CEN; *URA3* ; *$RAD27_{prom}$-RAD27*	Supplementary Fig. 4a	This study (e).
pBP2161	pRS313-TetOFF-YAT1	AmpR; CEN; *HIS3*; *CMV_{prom}-TetR-VP16, $tetO_7$- $CYC1_{prom}$-YAT1*	Fig. 2k	This study (f)
pBP2265	pET28a-RAD27	KanR; *$T7_{prom}$-RAD27-6xHis*	Fig. 3b-e, Supplementary Fig. 5a	This study (g).
pBP2266	pET28a-RAD27-D179A	KanR; *$T7_{prom}$-RAD27-D179A-6xHis*	Fig. 3b-d, Supplementary Fig. 5a	This study (h).
pBP2267	pET28a-RAD27-E176A	KanR; *$T7_{prom}$-RAD27-E176A-6xHis*	Fig. 3b-d, Supplementary Fig. 5a	This study (h).
pBP2146	pRS316-RAD27-E176A	AmpR; CEN; *URA3* ; *$RAD27_{prom}$-RAD27E176A*	Fig. 3f, Supplementary Fig. 5b	This study (h).
pBP2141	pRS425-EXO1	AmpR; 2μ; *LEU2* ; *$ADH1_{prom}$-EXO1*	Fig. 3g-h, Supplementary Fig. 5c	This study (i).
pBP1751	pRS316-GAL-YAT1	AmpR; CEN ; *URA3* ; *$GAL1_{prom}$-YAT1*	Supplementary Fig. 6a-b	Bonnet et al.[15]
pBP1789	pRS316-L-YAT1	AmpR; CEN ; *URA3* ; *$LEU2_{prom}$-leu2Δ3'-$GAL1_{prom}$-YAT1-leu2Δ5'*	Fig. 4d, j, Supplementary Fig. 6c, 7d	Bonnet et al.[9]
pBP1790	pRS316-L-intron-YAT1	AmpR; CEN ; *URA3* ; *$LEU2_{prom}$-leu2Δ3'-$GAL1_{prom}$-RPL51A*intron-YAT1-leu2Δ5'*	Fig. 4d, Supplementary Fig. 6c	Bonnet et al.[9]
pBP1932	pRS423-GPD-hsRNH1	AmpR; 2μ*; HIS3; GPD_{prom}-myc-hsRNH1-$CYC1_{term}$*	Fig. 4f, k	Bonnet et al.[9]
pWJ1344	pRS415-RAD52-YFP	AmpR ; CEN ; *LEU2* ; *$RAD52_{prom}$-RAD52-YFP*	Fig. 4k	Alvaro et al.[1]

(a) The *yfp-LYS2* reporter cassette encompasses: (i) the *ADH1* promoter; (ii) a non-functional *yfp* CDS carrying a 229bp 3' deletion; (iii) the *LYS2* gene under the control of its natural promoter; (iv) a non-functional *yfp* CDS harboring a frameshift 6bp downstream of the start codon (*5'fs-yfp*); the *ADH1* terminator. Both *yfp* fragments share a 500bp region of homology and SSA-mediated recombination using these repeated regions generate a functional *YFP* gene.

(b) The *leu2-YAT1* reporter cassette encompasses: (i) the *LEU2* promoter; (ii) a truncated *LEU2* CDS carrying a 345bp 3' deletion (*leu2Δ3'*); (iii) the *YAT1* gene under the control of the *GAL1-10* promoter; (iv) a hygromycin resistance marker (*TEF_{prom}-hphMX6-TEF_{term}* from *pFA6a-HphMX6*); (v) a truncated *LEU2* CDS carrying a 152bp 5' deletion (*leu2Δ5'*); (vi) the natural terminator of the *LEU2* gene. Both *LEU2* fragments share a 558bp region of homology and SSA-mediated recombination using these repeated regions generate a functional *LEU2* gene.

(c) The plasmid construct for stable integration of RHINO into the AAVS1 safe harbour locus of MCF7 breast cancer human cell lines was made by fusing the RHINO construct described before[12] with a TRE3GS promoter consisting of a minimal RNA Polymerase II binding sequence with 7 Tet operator binding sites from the pLVX-TetOne-Puro plasmid (Clontech). Next, the TRE3GS-RHINO fragment was ligated into into the pMK232 (CMV-OsTIR1-PURO) plasmid, a gift from Masato Kanemaki (Addgene plasmid #72834[16]), thereby replacing the CMV promoter and OsTIR sequences. Following this, a fragment from pLVX-TetOne-Puro containing the TetOn(R)3G transcriptional activator under the control of a human PGK promoter and the Puromycin resistance gene under the control of a SV40 promoter were ligated into the pMK232-TRE3GS-RHINO construct with homology arms for the human AAVS1 locus to make the final pMK232-TRE3GS-RHINO-TetON-Puro construct.

(d) RNase H1 constructs were generated by cloning the human RNase H1 short (nuclear) version CDS from ppyC AG_RNase H1_WT or ppyC AG_RNase H1_D210N (a gift from Xiang Dong Fu, Addgene plasmid #111906 and #11904[17]) into pUBC-GFP-C1 or pUBC-mScarlet-i-C1 vectors.

(e) A fragment encompassing *RAD27* complete CDS, together with 1kb of 5' and 500bp of 3' flanking regions, was cloned into pRS316. This construct allows expression of *RAD27* under the control of its natural promoter and terminator sequences.

(f) The *TetOFF-YAT1* reporter construct was obtained by inserting $\mathit{CMV_{prom}}$*-TetR-VP16* and $\mathit{tetO_7}$-$\mathit{CYC1_{prom}}$ cassettes[18] upstream *YAT1* CDS.

(g) *RAD27 c*omplete CDS was cloned into pET28a (Novagen), as a C-terminal fusion with a poly-Histidine tag, allowing recombinant protein expression and purification.

(h) Obtained by site-directed mutagenesis of above-described constructs.

(i) *EXO1* complete CDS was cloned downstream the strong, constitutive *ADH1* promoter, allowing *in vivo* over-expression.

Supplementary Table 3 (Oligonucleotides for real-time PCR, synthetic spike-in and *in vitro* cleavage assays)

NAME	SEQUENCE	USAGE
PMA1 F	TTGCCAGCTGTCGTTACCAC	Real-time PCR
PMA1 R	TCGACACCAGCCAAGGATTC	Real-time PCR
YEF3 F	GATTGCCGGTGGTAAGAAGA	Real-time PCR
YEF3 R	CGTAAGCATCACCCAATTCC	Real-time PCR
ADH1 F	TCTTCGCCAGAGGTTTGGTC	Real-time PCR
ADH1 R	CCAACGATTTGACCCTTTTCCA	Real-time PCR
YAT1 F	TCTGTGGTGGTGTCCTCAAG	Real-time PCR
YAT1 R	CTTGCTGCCGTTTGAAGATG	Real-time PCR
Intergenic 1 F	GAAACCACGAAAAGTTCACCA	Real-time PCR
Intergenic 1 R	AGCTTCTGCAAACCTCATTTG	Real-time PCR
Intergenic 2 F	CGCATTACCAGACGGAGATGT	Real-time PCR
Intergenic 2 R	CAAGCAAGCCTTGTGCATAAGA	Real-time PCR
SpikeIn F	AGAAACGTGCACTTGGCATT	Real-time PCR
SpikeIn R	GCAGCTGGGTGTGTATTTTG	Real-time PCR
SpikeIn-DNA-F	CTATATGCAGCTGGGTGTGTATTTTGTAAACAGAAGTAATTTTCAACTTCTAAG CTTTGTATACAAAGCACTGCCGTAGCAATGCCAAGTGCACGTTTCT	Spike-in for DRIP-seq and DRIP-qPCR experiments
SpikeIn-RNA-R	rArGrArArArCrGrUrGrCrArCrUrUrGrGrCrArUrUrGrCrUrArCrGrGrCrArGrUrGrCrUrU rUrGrUrArUrArCrArArArGrCrUrUrArGrArArGrUrUrGrArArArArUrUrArCrUrUrCrUrG rUrUrUrArCrArArArArUrArCrArCrArCrCrCrArGrCrUrGrCrArUrArUrArG	Spike-in for DRIP-seq and DRIP-qPCR experiments
common-90-R	GTCACTTGATAAGAGGTCATTTGAACACTCACGCACCGACTCTAGCCCTAACG ACTCAGACCACGTCCAACATGTTTTAAATATGCAATG	*In vitro* cleavage assays (R-loop and flap substrates)
[FLO]-R-loop-90-F	[FLO]CATTGCATATTTAAAACATGTTGGATTGAATGCATGGCTTAGATCTGAAT TGCTGAGTCTGGTGCTTCAAATGACCTCTTATCAAGTGAC	*In vitro* cleavage assays (R-loop substrate)
[Cy5]-R-loop-RNA-F	[Cy5]rCrGrUrGrGrUrCrUrGrArGrUrCrGrUrUrArGrGrGrCrUrArGrArGrUrCrGrGrUrGr CrGrUrGrArGrUrG	*In vitro* cleavage assays (R-loop substrate)
[FLO]-Flap-65-F	[FLO]TTGAATGCATGGCTTAGATCTGAATTGCTGAGTCTGGTGCTTCAAATGAC CTCTTATCAAGTGAC	*In vitro* cleavage assays (flap substrate)
Flap-66-F	CATTGCATATTTAAAACATGTTGGACGTGGTCTGAGTCGTTAGGGCTAGAGTCG GTGCGTGAGTGC	*In vitro* cleavage assays (flap substrate)

Supplementary references

1. Alvaro, D. *et al.* Systematic hybrid LOH: a new method to reduce false positives and negatives during screening of yeast gene deletion libraries. *Yeast* **23**, 1097–1106 (2006).
2. Wahba, L., Costantino, L., Tan, F. J., Zimmer, A. & Koshland, D. S1-DRIP-seq identifies high expression and polyA tracts as major contributors to R-loop formation. *Genes Dev* **30**, 1327–38 (2016).
3. Aiello, U. *et al.* Sen1 is a key regulator of transcription-driven conflicts. *Mol. Cell* **82**, 2952-2966.e6 (2022).
4. Pelechano, V., Chávez, S. & Pérez-Ortín, J. E. A complete set of nascent transcription rates for yeast genes. *PloS One* **5**, e15442 (2010).
5. McGuffee, S. R., Smith, D. J. & Whitehouse, I. Quantitative, genome-wide analysis of eukaryotic replication initiation and termination. *Mol. Cell* **50**, 123–135 (2013).
6. Reid, R. J. D. *et al.* Chromosome-scale genetic mapping using a set of 16 conditionally stable Saccharomyces cerevisiae chromosomes. *Genetics* **180**, 1799–1808 (2008).
7. Reid, R. J. D. *et al.* Selective ploidy ablation, a high-throughput plasmid transfer protocol, identifies new genes affecting topoisomerase I-induced DNA damage. *Genome Res.* **21**, 477–486 (2011).
8. Li, Z. *et al.* Systematic exploration of essential yeast gene function with temperature-sensitive mutants. *Nat Biotechnol* **29**, 361–7 (2011).
9. Bonnet, A. *et al.* Introns Protect Eukaryotic Genomes from Transcription-Associated Genetic Instability. *Mol. Cell* **67**, 608-621.e6 (2017).
10. Lisby, M., Rothstein, R. & Mortensen, U. H. Rad52 forms DNA repair and recombination centers during S phase. *Proc Natl Acad Sci U A* **98**, 8276–82 (2001).
11. Hentges, P., Van Driessche, B., Tafforeau, L., Vandenhaute, J. & Carr, A. M. Three novel antibiotic marker cassettes for gene disruption and marker switching in Schizosaccharomyces pombe. *Yeast* **22**, 1013–9 (2005).
12. Martin, R. M., de Almeida, M. R., Gameiro, E. & de Almeida, S. F. Live-cell imaging unveils distinct R-loop populations with heterogeneous dynamics. *Nucleic Acids Res.* **51**, 11010–11023 (2023).
13. Vítor, A. C. *et al.* Single-molecule imaging of transcription at damaged chromatin. *Sci. Adv.* **5**, eaau1249 (2019).
14. Amon, J. D. & Koshland, D. RNase H enables efficient repair of R-loop induced DNA damage. *eLife* **5**, e20533 (2016).
15. Bonnet, A., Bretes, H. & Palancade, B. Nuclear pore components affect distinct stages of intron-containing gene expression. *Nucleic Acids Res* **43**, 4249–61 (2015).
16. Natsume, T., Kiyomitsu, T., Saga, Y. & Kanemaki, M. T. Rapid Protein Depletion in Human Cells by Auxin-Inducible Degron Tagging with Short Homology Donors. *Cell Rep.* **15**, 210–218 (2016).
17. Chen, L. *et al.* R-ChIP Using Inactive RNase H Reveals Dynamic Coupling of R-loops with Transcriptional Pausing at Gene Promoters. *Mol. Cell* **68**, 745-757.e5 (2017).
18. Garí, E., Piedrafita, L., Aldea, M. & Herrero, E. A set of vectors with a tetracycline-regulatable promoter system for modulated gene expression in Saccharomyces cerevisiae. *Yeast Chichester Engl.* **13**, 837–848 (1997).
