# Supplementary material for: DNA lesions can frequently precede DNA:RNA hybrid accumulation
Source: Nat Commun. 2025 Mar 10;16:2401. doi: 10.1038/s41467-025-57588-x (PMC11893903; doi:10.1038/s41467-025-57588-x)
Supplement: Supplementary file 2 — Reporting Summary [file 41467_2025_57588_MOESM2_ESM.pdf]

nature portfolio

Corresponding author(s): Benoit Palancade

Last updated by author(s): Feb 10, 2025

Reporting Summary

Nature Portfolio wishes to improve the reproducibility of the work that we publish. This form provides structure for consistency and transparency in reporting. For further information on Nature Portfolio policies, see our Editorial Policies and the Editorial Policy Checklist.

Statistics

For all statistical analyses, confirm that the following items are present in the figure legend, table legend, main text, or Methods section.

n/a	Confirmed	
☐	☒	The exact sample size (*n*) for each experimental group/condition, given as a discrete number and unit of measurement
☐	☒	A statement on whether measurements were taken from distinct samples or whether the same sample was measured repeatedly
☐	☒	The statistical test(s) used AND whether they are one- or two-sided<br>*Only common tests should be described solely by name; describe more complex techniques in the Methods section.*
☒	☐	A description of all covariates tested
☒	☐	A description of any assumptions or corrections, such as tests of normality and adjustment for multiple comparisons
☐	☒	A full description of the statistical parameters including central tendency (e.g. means) or other basic estimates (e.g. regression coefficient) AND variation (e.g. standard deviation) or associated estimates of uncertainty (e.g. confidence intervals)
☒	☐	For null hypothesis testing, the test statistic (e.g. *F*, *t*, *r*) with confidence intervals, effect sizes, degrees of freedom and *P* value noted<br>*Give P values as exact values whenever suitable.*
☒	☐	For Bayesian analysis, information on the choice of priors and Markov chain Monte Carlo settings
☒	☐	For hierarchical and complex designs, identification of the appropriate level for tests and full reporting of outcomes
☒	☐	Estimates of effect sizes (e.g. Cohen's *d*, Pearson's *r*), indicating how they were calculated

Our web collection on statistics for biologists contains articles on many of the points above.

Software and code

Policy information about availability of computer code

Data collection	n.a.
Data analysis	n.a.

For manuscripts utilizing custom algorithms or software that are central to the research but not yet described in published literature, software must be made available to editors and reviewers. We strongly encourage code deposition in a community repository (e.g. GitHub). See the Nature Portfolio guidelines for submitting code & software for further information.

Data

Policy information about availability of data

All manuscripts must include a data availability statement. This statement should provide the following information, where applicable:

- Accession codes, unique identifiers, or web links for publicly available datasets
- A description of any restrictions on data availability
- For clinical datasets or third party data, please ensure that the statement adheres to our policy

The complete NGS data generated during this study are available in the Gene Expression Omnibus (GEO) database under accession number GSE265894 (DRIP-seq) and GSE265892 (RNA-seq). All the other data supporting the findings of this study are available within the paper and its Supplementary Information. Source Data for all figures are provided with this paper.

Research involving human participants, their data, or biological material

Policy information about studies with human participants or human data. See also policy information about sex, gender (identity/presentation), and sexual orientation and race, ethnicity and racism.

Reporting on sex and gender	n.a.
Reporting on race, ethnicity, or other socially relevant groupings	n.a.
Population characteristics	n.a.
Recruitment	n.a.
Ethics oversight	n.a.

Note that full information on the approval of the study protocol must also be provided in the manuscript.

Field-specific reporting

Please select the one below that is the best fit for your research. If you are not sure, read the appropriate sections before making your selection.

☒ Life sciences ☐ Behavioural & social sciences ☐ Ecological, evolutionary & environmental sciences

For a reference copy of the document with all sections, see nature.com/documents/nr-reporting-summary-flat.pdf

Life sciences study design

All studies must disclose on these points even when the disclosure is negative.

Sample size	No statistical methods were used to predetermine sample sizes ; (n) values were chosen in accordance with standard practices and correspond to the number of biological replicates (e.g. independent yeast cultures) performed in each situation.
Data exclusions	None samples were excluded.
Replication	Experiments were systematically replicated and all replicates were successfully giving similar results.
Randomization	The experiments were not randomized.
Blinding	The investigators were not blinded to allocation during experiments and outcome assessment.

Reporting for specific materials, systems and methods

We require information from authors about some types of materials, experimental systems and methods used in many studies. Here, indicate whether each material, system or method listed is relevant to your study. If you are not sure if a list item applies to your research, read the appropriate section before selecting a response.

Materials & experimental systems

n/a	Involved in the study	
☐	☒	Antibodies
☐	☒	Eukaryotic cell lines
☒	☐	Palaeontology and archaeology
☒	☐	Animals and other organisms
☒	☐	Clinical data
☒	☐	Dual use research of concern
☒	☐	Plants

Methods

n/a	Involved in the study	
☐	☒	ChIP-seq
☐	☒	Flow cytometry
☒	☐	MRI-based neuroimaging

Antibodies

Antibodies used	Antibodies' manufacturers and references are indicated in the METHODS section: anti DNA:RNA hybrids (S9.6, Kerafast; Cat# ENH001; RRID:AB_2687463), anti double-stranded DNA (HYB331-01, Santa Cruz Biotechnology; Cat# sc-58749; RRID: AB_7830 88), anti-Flag monoclonal antibody (M2, Sigma; Cat# F1804; RRID:AB_262044), anti-Dpm1 monoclonal antibody (Thermo Fisher Scientific; Cat# A6429; RRID:AB_2536204), anti RNAP II largest subunit (anti-Rpb1, BioLegend; Cat# 920102), anti-phosphoH2A.X antibodies (Millipore, Cat# 05-636), Alexa Fluor 568 goat anti-mouse antibodies (Invitrogen; Cat# A-11004), Alexa Fluor 647 goat anti-mouse antibodies (Thermo Fisher Scientific; Cat# A-21236, Peroxidase AffiniPure goat anti-mouse IgG+IgM (Jackson

	ImmunoResearch; Cat# 115-035-068; RRID: AB_2338505).
Validation	The antibodies used in this study were previously validated (see manufacturer's websites or references in the METHODS section).

Eukaryotic cell lines

Policy information about cell lines and Sex and Gender in Research

Cell line source(s)	MCF7: ATCC, Cat# ATCCHTB-22 (female).
Authentication	No authentification ; stable cell lines were isolated by sorting based on mNeonGreen transgene expression.
Mycoplasma contamination	Tested negative for mycoplasma contamination.
Commonly misidentified lines (See ICLAC register)	n.a.

Plants

Seed stocks	n.a.
Novel plant genotypes	n.a.
Authentication	n.a.

ChIP-seq

Data deposition

- [x] Confirm that both raw and final processed data have been deposited in a public database such as GEO.
- [ ] Confirm that you have deposited or provided access to graph files (e.g. BED files) for the called peaks.

Data access links<br>*May remain private before publication.*	Featured experiments are DNA:RNA hybrid immunoprecipitations coupled to sequencing (DRIP-seq). Data are available in the Gene Expression Omnibus (GEO) database under accession number GSE265894.
Files in database submission	GSM8231036 yeast, Eluate, IAA G1, rep1<br>GSM8231037 yeast, Eluate, IAA, rep1<br>GSM8231038 yeast, Eluate, IAA, rep2<br>GSM8231039 yeast, Eluate, noIAA, rep1<br>GSM8231040 yeast, Eluate, noIAA, rep2<br>GSM8231041 yeast, Input, IAA G1, rep1<br>GSM8231042 yeast, Input, IAA, rep1<br>GSM8231043 yeast, Input, noIAA, rep1<br>GSM8231044 yeast, Eluate Rnh, IAA G1, rep1<br>GSM8231045 yeast, Eluate Rnh, IAA, rep1<br>GSM8231046 yeast, Eluate Rnh, noIAA, rep1
Genome browser session (e.g. UCSC)	Bigwig files have been provided within the GEO dataset for uploading on genome browsers.

Methodology

Replicates	Two replicates were performed for each strain/condition (unsynchronized cells); DRIP-qPCR (n=4) was used to validate hits from DRIP-seq.
Sequencing depth	GSM8231036 yeast, Eluate, IAA G1, rep1; 28135295 total reads; 14233067 uniquely mapped reads, paired-end reads 2x75bp<br>GSM8231037 yeast, Eluate, IAA, rep1; 22885909 total reads; 8693467 uniquely mapped reads, paired-end reads 2x75bp<br>GSM8231038 yeast, Eluate, IAA, rep2; 27043390 total reads; 10123331 uniquely mapped reads, paired-end reads 2x75bp<br>GSM8231039 yeast, Eluate, noIAA, rep1 26969227 total reads; 14693669 uniquely mapped reads, paired-end reads 2x75bp<br>GSM8231040 yeast, Eluate, noIAA, rep2; 24997419 total reads; 13326529 uniquely mapped reads, paired-end reads 2x75bp<br>GSM8231041 yeast, Input, IAA G1, rep1; 26848433 total reads; 15270347 uniquely mapped reads, paired-end reads 2x75bp<br>GSM8231042 yeast, Input, IAA, rep1; 26820076 total reads; 17760446 uniquely mapped reads, paired-end reads 2x75bp<br>GSM8231043 yeast, Input, noIAA, rep1; 16028723 total reads; 10572576 uniquely mapped reads, paired-end reads 2x75bp<br>GSM8231044 yeast, Eluate Rnh, IAA G1, rep1; 7549766 total reads; 1903925 uniquely mapped reads, paired-end reads 2x75bp

	GSM8231045 yeast, Eluate Rnh, IAA, rep1; 26897692 total reads; 9983547 uniquely mapped reads, paired-end reads 2x75bp<br>GSM8231046 yeast, Eluate Rnh, noIAA, rep1; 26993323 total reads; 9614803 uniquely mapped reads, paired-end reads 2x75bp
Antibodies	anti DNA:RNA hybrids (S9.6, Kerafast; Cat# ENH001; RRID:AB_2687463).
Peak calling parameters	No peak calling was performed for the analysis of DRIP-seq datasets (hence no BED files provided for called peaks).
Data quality	Signal specificity was assessed following in vitro RNAse H treatment.
Software	Bowtie2, SAMTools and BEDTools in GALAXY (usegalaxy.org).

Flow Cytometry

Plots

Confirm that:

- [x] The axis labels state the marker and fluorochrome used (e.g. CD4-FITC).
- [x] The axis scales are clearly visible. Include numbers along axes only for bottom left plot of group (a 'group' is an analysis of identical markers).
- [ ] All plots are contour plots with outliers or pseudocolor plots.
- [ ] A numerical value for number of cells or percentage (with statistics) is provided.

Methodology

Sample preparation	For assessing YFP expression, live yeast cells were harvested and directly analyzed. For validating cell synchronization and release (Propidium Iodide staining following alpha-factor treatment), cells were fixed in 70% ethanol (1h, room temperature) and further treated with RNAse A (1 µg/µL, 1h, 37°C) and pepsin (0.5%, 30min, 37°C) prior to analysis.
Instrument	BD FacSCAN or BD FACSCalibur cytometers (YFP expression analysis; Fig. 1) ; BD Accuri C6 Plus (PI staining for validating cell synchronization; Extended Data Fig. 4c) ; Attune Cytpix Flow Cytometer (PI staining for analyzing release into the cell cycle; Extended Data Fig. 4e).
Software	OMIQ (Dotmatics)
Cell population abundance	No cell populations were sorted for further experimental analysis.
Gating strategy	FSC/SSC gates were used to identify viable cells. SSC-A/SSC-H gates were used to exclude cell doublets and aggregates. Gating used as references (i) control non-fluorescent strains containing empty vector plasmids (YFP expression analysis) or (ii) unlabeled cells (PI staining for validating cell synchronization).

- [x] Tick this box to confirm that a figure exemplifying the gating strategy is provided in the Supplementary Information.
