# Supplementary material for: DNA lesions can frequently precede DNA:RNA hybrid accumulation
Source: Nat Commun. 2025 Mar 10;16:2401. doi: 10.1038/s41467-025-57588-x (PMC11893903; doi:10.1038/s41467-025-57588-x)
Supplement: Supplementary file 3 — Description of Supplementary Data files [file 41467_2025_57588_MOESM3_ESM.pdf]

Description of Additional Supplementary Files

File name: Supplementary Data 1

Description: Results of the hyper-rec and hybrid screens
